# Endothelial Cells Activated by Extracellular Histones Promote Foxp3^+^ Suppressive Treg Cells In Vitro

**DOI:** 10.3390/ijms23094527

**Published:** 2022-04-20

**Authors:** Marine Arnaud, Jordane Demonchy, Eden Arrii, Marta Luperto, Julien Lion, Sofiane Fodil, Stéphanie Pons, Nuala Mooney, Lara Zafrani

**Affiliations:** 1 Human Immunology, Pathophysiology and Immunotherapy, INSERM U 976, University Paris Cite, 75010 Paris, France; marine.arnaud@inserm.fr (M.A.); jordane.demonchy@gmail.com (J.D.); edenarrii@gmail.com (E.A.); marta.luperto01@gmail.com (M.L.); julien.lion@inserm.fr (J.L.); sofinae.fodil@aphp.fr (S.F.); pons.stephanie0@gmail.com (S.P.); nuala.mooney@univ-paris-diderot.fr (N.M.); 2 Medical Intensive Care Unit, Assistance Publique des Hôpitaux de Paris, Saint Louis Hospital, 75010 Paris, France

**Keywords:** extracellular histones, endothelial cells, T regulatory lymphocytes

## Abstract

Histones are widely recognized as pro-inflammatory mediators upon their release from the nucleus into the extracellular space. However, their impact on endothelial cell immunogenicity is unknown. Endothelial cells, Human Microvascular Endothelial cells 1 (HMEC1), have been exposed to recombinant histones in order to study their effect on the endothelial phenotype. We then studied the differentiation of CD4^+^-T lymphocytes subpopulations after three days of interaction with endothelial cells in vitro and observed that histone-treated endothelial cells differentiate a suppressive FoxP3^+^ T regulator subpopulation that expressed Human Leucocyte Antigen DR (HLA-DR) and Cytotoxic T-Lymphocyte-Associated protein 4 (CTLA4). Toll-Like Receptor 4 (TLR4) inhibition significantly decreased the expansion of these Treg cells. Moreover, blockade of Interleukin (IL)-6 and Intercellular Adhesion Molecule (ICAM)-1 in cocultures significantly decreased the expansion of Tregs, suggesting an IL-6 and ICAM-1 dependent pathway. Thus, beyond their inflammatory effects, extracellular histones may induce an increase of immunosuppressive Treg population via their action on endothelial cells. Further studies are needed to evaluate the impact on immunosuppression of an increase of peripheral suppressive Treg via endothelial cell activation by histones in vivo.

## 1. Introduction

The vascular endothelium forms an active interface between blood and tissues and thereby participates in multiple organ dysfunction during sepsis [1], trauma [2] or sepsis-like syndrome, such as tumor lysis syndrome [3]. Accumulating evidence has demonstrated that endothelial cells are targets in these pathologies, and they also play a crucial role in mediating immune responses. Indeed, human endothelial cells constitutively express Human Leucocyte Antigen (HLA)-I and, depending on their location, also express low-level HLA-II that is strongly upregulated by inflammation. They also express co-stimulatory and co-inhibitory molecules allowing them to activate and mediate differentiation of CD4^+^-T lymphocyte subpopulations [4]. Among these subpopulations endothelial cells can expand functional CD4^+^ T regulatory cells (Tregs) in an inflammatory setting. This population can contribute to suppression of immune response in pathologies such as sepsis [5] or tumor malignancies [6].

When cell death is extensive—for example, in tumor lysis syndrome induced by chemotherapy in strongly proliferative malignancies—high levels of unchained extracellular histones are released and convey endothelial cytotoxic effects [7]. Histones are widely recognized as pro-inflammatory mediators upon their release from the nucleus into the extracellular space. Histones consist of five cationic proteins in the nucleus of eukaryotic cells. In the context of sepsis [7], trauma [8], or tumor lysis syndrome, some authors have suggested that extracellular release of histones may contribute to endothelial dysfunction [9,10,11,12,13,14,15].

This study shows that human endothelial cells activated by recombinant histones produce pro-inflammatory cytokines, increase adhesion molecule expression and induce, by a mechanism implicating InterCellular Adhesion Molecule (ICAM)-1 and InterLeukin (IL)-6 secretion, the proliferation of a functionally suppressive CD25^high^ FoxP3^high^ HLA-DR^+^ Treg cell population without altering pro-inflammatory CD4^+^ T cells.

## 2. Results

### 2.1. Effect of Extracellular Histones on Endothelial Secretion of Pro-Inflammatory Cytokines and Expression of Adhesion Molecules

**Figure 1:**
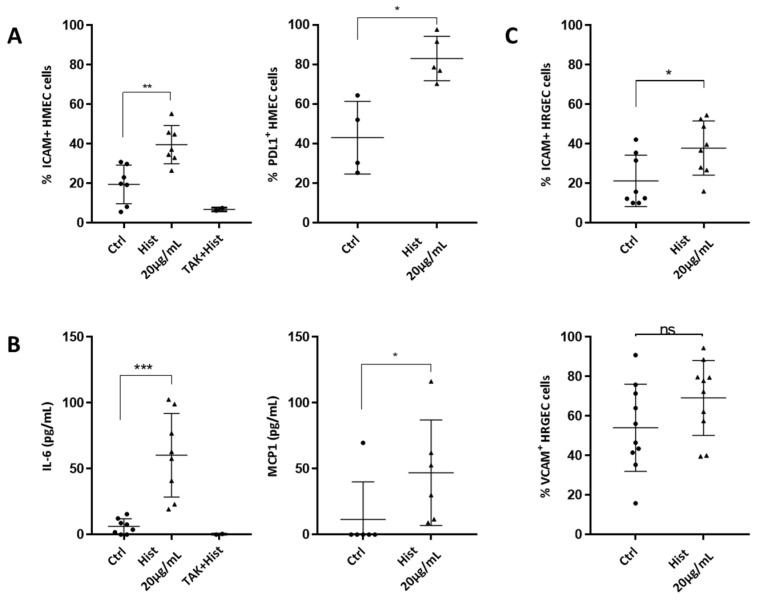
Histones induce a pro-inflammatory activation of endothelial cells. (**A**) ICAM-1 and PDL1 were assessed in HMEC cells activated with 20 µg/mL of recombinant histones. The percentage of positive cells was evaluated by cytometry. Histones significantly increased ICAM-1 and PDL-1 expression (Mann–Whitney test, ** *p* = 0.002, * *p* = 0.016, n = 5–7), and this was abrogated by TLR4 inhibition. (**B**) HMEC cells were seeded and then cultured for 18 h with the noncytotoxic dose of 20 µg/mL histone, before measuring pro-inflammatory cytokines and chemokines in the culture supernatants by ELISA. Levels of IL-6, MCP1, IL-10 and IL-2 were evaluated. Neither IL-10 nor IL-2 was detected whereas both IL-6 and MCP-1 were significantly increased (Mann–Whitney test, *** *p* = 0.0002, * *p* = 0.035, n = 6–8). The increase in IL-6 was reversed by TLR4 inhibition. (**C**) ICAM-1 expression was increased in HRGEC cells activated with 20 µg/mL of recombinant histones. The percentage of positive cells was evaluated by cytometry (unpaired *t*-test * *p* = 0.026, n = 8–10). The expression of VCAM-1 was not significantly increased under these conditions.

In order to investigate the role of extracellular histones on endothelial cell activation, Human Microvascular Endothelial Cells 1 (HMEC1s) and Human Renal Glomerular Endothelial Cells (HRGECs) were exposed for 18 h to 20 µg/mL of extracellular histones, the noncytotoxic concentration defined in our prior study [16]. After stimulation, we investigated adhesion molecule expression and we found that Intercellular Adhesion Molecule-1 (ICAM-1) expression was increased on HMEC1s (Figure 1A) and HRGECs (Figure 1C). Programmed cell Death Ligand 1 (PDL1) was also increased in HMEC1 cells by histones (Figure 1A). Moreover, secretion of IL-6, and Monocyte Chemoattractant Protein 1 (MCP-1) were significantly increased by histone administration (Figure 1B). Histones did not increase either HLA-I or HLA-DR expression on HMECs (Supplementary Table S1).

In order to study potential differences between individual histones in the activation of endothelial cells, we examined the impact of H1, H2A, H3 and H4 on HMECs. H3 and H4 induced HMECs activation with significant increased expression of ICAM-1 and increased secretion of IL-6 at 20 µg/mL. H1 and H2A increased neither the expression of ICAM-1 nor IL-6 secretion (Supplementary Figure S1).

Previous studies showed that histones specifically bind to Toll-Like Receptor (TLR)4 on endothelial cells [17]. Blocking the TLR4 pathway by a 1-h pre-incubation with TAK242 significantly inhibited histone-activated ICAM1 expression and IL6 secretion by endothelial cells (Figure 1A,B). In contrast, inhibition of TLR2 failed to decrease either ICAM-1 or PDL-1 expression or IL-6 secretion in HMECs activated by histones (Supplementary Figure S2).

### 2.2. Induction of a Treg Population by Histone-Treated HMEC

**Figure 2:**
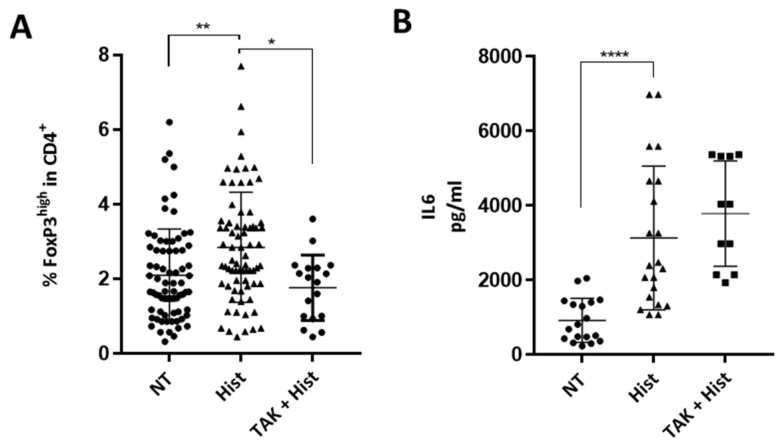
Endothelial activation mediated by histones induce Treg proliferation. (**A**) Comparison of Treg expansion after three days of coculture, with HMECs, pre-stimulated or not with histones, and with or without TAK 242 pre-incubation. Results are presented as the percentage of CD4^+^ CD25^+^ CD127^low^ FoxP3^high^ among CD4^+^ cells. Gating was performed using Fluorescence Minus One (FMO) control. Histone pre-treatment increased Treg expansion (Kruskal–Wallis test, ** *p* = 0.002), and this was strongly reduced by TLR4 inhibition (Kruskal-Wallis test, * *p* = 0.011, (n = 18–73)). (**B**) IL-6 was measured in co-culture supernatants. Histone pre-treatment increased IL-6 secretion in co-culture, and this was not reversed by TLR4 inhibition (Kruskal–Wallis test, **** *p* < 0.0001 (n = 12–23)).

To investigate whether histones alter endothelial cell immunogenicity, endothelial cells pre-activated by histones were co-cultured with Peripheral Blood Mononuclear Cells (PBMCs) for three days, and CD4^+^ T cell polarization was evaluated. Endothelial cells activated by histones significantly increased the expansion of a FoxP3^+^ Treg cell population in comparison with the nontreated condition (Figure 2A). The PBMCs used for these experiments were obtained from healthy blood donors attending the local blood transfusion service. Forty-seven donors were male and twenty-five were female, with a median age of 35 years old (IQR 29-49). Treg expansion after histone pre-stimulation of HMECs were similar according to sex and age of the donors (Supplementary Figure S3). Inhibition of TLR4 by pre-incubation with TAK-242 significantly decreased expansion of Treg cells (Figure 2A). Representative gating of the Treg population is shown in Supplementary Figure S4. In contrast, there was no difference in the differentiation of Th17 or Th1 cells (Supplementary Figure S5A). Representative gating of the Th1 and Th17 population is found in Supplementary Figure S5B.

Moreover, IL-6 was strongly increased in PBMC-HMEC co-cultures after exposure of HMECs to histones, and this was unaltered by TLR4 inhibition (Figure 2B). Because IL-2 is crucial for the maintenance of Tregs, we also tested IL-2 levels in co-culture supernatants but did not detect IL-2 at the end of a three-day co-culture. IL-6 secretion in PBMC-HMEC interactions was not altered by TLR4 inhibition (Figure 2B).

### 2.3. Characterization of Histone-Induced Treg Cells

**Figure 3:**
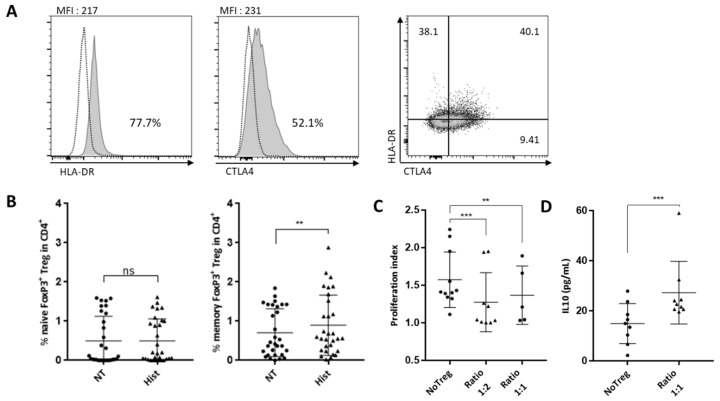
Phenotype and function of Treg induced by histone-activated endothelial cells. (**A**) Phenotype of Tregs induced in cocultures with HMEC pre-treated with histones were assessed using anti-CD45-RA, -HLA-DR and -CTLA-4 antibodies. Gating was performed using Fluorescence Minus One (FMO) control. (**B**) Naïve Tregs are CD45RA^+^ whereas CD45RA^−^ are memory Tregs. Results are expressed as the percentage of cells in CD4^+^ cells. Memory Treg expansion was significantly increased in co-culture with histone-pretreated endothelial cells (Wilcoxon test, ** *p* = 0.0037, n = 30). (**C**) The suppressive ability of Tregs sorted from co-cultures with HMECs pre-treated with histones was assessed. Proliferation of autologous CD4^+^-T cells were measured by dilution of Cell Trace Violet staining. Analysis was made with the Proliferation Tool on FlowJo X. The proliferation index from three different conditions is reported here: Tresp alone, ratio 1 Treg:2 Tresp, and ratio 1 Treg:1 Tresp. Proliferation Index is the total number of cell divisions divided by the number of cells that went into division. Adapted with permission from FlowJo Tutorial, Tech Notes and Web Site Copyright © FlowJo LLC., 1997–2019 [18]. Tregs significantly reduced CD4^+^-T cells proliferation at the ratio 1:2 (Paired *t*-test, *** *p* = 0.0007) and at the ratio 1:1 (Paired *t*-test, ** *p* = 0.0052, (n = 5–9)). (**D**) Levels of IL-10 were measured by ELISA at the end of the suppression assay. IL-10 concentration was increased in the presence of Tregs (Mann–Whitney test, *** *p* = 0.001, n = 9).

In order to further explore the phenotype and function of Tregs generated in response to histone mediated activation of endothelial cells, Cytotoxic T-Lymphocyte-Associated protein 4 (CTLA-4), HLA-DR and retinoic-acid-receptor-related orphan nuclear receptor gamma (RORyt) expression in Tregs were studied after PBMC interaction with HMEC that had been pre-activated with histones or not. Seventy-eight percent of CD25^high^ FoxP3^high^ Treg cells expressed HLA-DR and more than half of CD25^high^ FoxP3^high^ Treg cells expressed CTLA4 when endothelial cells had been pre-exposed to histones (Figure 3A). CTLA-4 and HLA-DR were co-expressed on 40% of Treg cells (Figure 3A). CD45RA expression was determined in Treg cells (gating in Supplementary Figure S4) and revealed that this population was CD45RA^low^ (Figure 3B) and therefore memory T cells. Although a RORyt expressing Treg population has been reported (Ref), RORyt expression was not detected in Treg induced by histone-activated endothelial cells (Supplementary Figure S5A).

As phenotype alone is not an exact indicator of function, we next tested whether Treg induced by endothelial cells activated by extracellular histones were functional. We determined whether Tregs inhibited proliferation of autologous T lymphocytes in response to anti-CD3/CD28 coated beads as previously described [19,20] and illustrated in Supplementary Figure S6. Isolated Treg subsets generated in endothelial cell co-cultures were added to autologous CD4^+^ T (designated as Tresponders, Tresp) that were activated by anti-CD3/CD28 coated beads at the indicated ratios. Tregs significantly reduced T effector proliferation at a ratio of 1 Treg for 2 Tresp (1:2) (proliferation index median of Tresp without Tregs = 1.41 versus 1.04 with Tregs) and 1 Treg for 1 Tresp (1:1) (proliferation index median of Tresp without Tregs = 1.21 versus 1.04 with Tregs) (Figure 3C).

Tregs can exert their suppressive activity via IL-10 secretion, so we measured IL-10 in the supernatant of the three-day suppression assay. IL-10 was two-fold higher in the Treg condition compared to controls without Treg (Figure 3D).

### 2.4. Interleukin 6 and ICAM-1 Are Both Involved in the Development of Suppressive Treg Induced by Histone-Treated HMECs

In view of the histone-induced increases of ICAM-1 and PDL1 expression and of IL-6 secretion by HMEC1 cells, we assessed whether ICAM-1, IL-6 and PDL1 were involved in the expansion of Tregs in our model. We treated histone-stimulated HMEC cells with anti-ICAM-1 or anti-IL-6 blocking antibodies prior to culturing them with PBMC.

**Figure 4:**
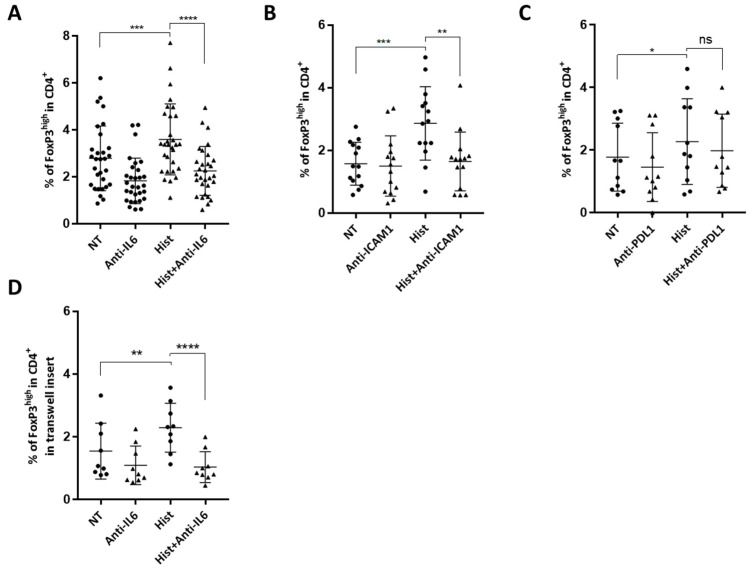
IL-6 and ICAM-1 are implicated in the mechanisms of Treg expansion in this model of endothelial cell activation by histones. (**A**) HMECs pre-treated with histones for 18 h were washed and treated with 10 ng/mL anti-IL-6 neutralizing antibody prior to adding PBMC. PBMC were stained after three days of co-culture to evaluate Treg expansion as described above. Results are presented as percentage of CD4^+^ CD25^+^ CD127^low^ FoxP3^high^ among CD4+ cells. Histone pre-treatment increased Treg expansion (Paired *t*-test, *** *p* = 0.0002, n = 30), and this was reduced after IL-6 blockade (Paired *t*-test, **** *p* < 0.0001, n = 30). (**B**) After histone exposure, HMECs were treated with 10 ng/mL anti-ICAM1 neutralizing antibody prior to co-culture with PBMC. PBMC were stained after three days to evaluate Treg expansion as described above. Results are presented as percentage of CD4^+^ CD25^+^ CD127^low^ FoxP3^high^ among CD4^+^ cells. Histone pre-treatment increased Treg expansion (Paired *t*-test, *** *p* = 0.0001, n = 12), and this was reduced with ICAM1 blockade (Paired *t*-test, ** *p* = 0.0012, n = 12). (**C**) After histone exposure, HMECs were treated with 10 ng/mL anti-PDL1 neutralizing antibody prior to co-culture with PBMC. PBMC were stained after three days to evaluate Treg expansion as described above. Results are presented as percentage of CD4^+^ CD25^+^ CD127^low^ FoxP3^high^ among CD4^+^ cells. Histone pre-treatment increased Treg expansion (Paired *t*-test, * *p* = 0.02, n = 11) and PDL-1 blockade did not significantly reduce Treg expansion. (**D**) Co-cultures were performed in Transwell^®^ inserts preventing cell contact, with or without pre-treatment with anti-IL6 antibody. Histone pre-treatment increased Treg expansion without cell-contact between endothelial cells and PBMCs (Paired *t*-test, ** *p* = 0.025, n = 9), and IL-6 blockage significantly reduced Treg expansion in this model (Paired *t*-test, **** *p* < 0.0001, n = 9).

When blocking antibodies directed against IL-6 or ICAM-1 were present, there was a significant decrease (38.5% for IL-6, and 40.8% for ICAM-1) in Tregs differentiated by endothelial cells stimulated with histones (Figure 4A,B). In contrast, blocking PDL1 did not significantly change the levels of Treg in co-cultures (Figure 4C).

To determine whether cell contact was required for Treg expansion, we performed cocultures in Transwell^®^ (Kennebunk, ME, USA) plates, preventing cell contact between PBMCs and HMECs. We showed that Tregs differentiation was maintained even in the absence of cell-contact in cocultures (Figure 4D). Interestingly, treatment with antibody blocking IL-6 in these conditions decreased the expansion of Treg cells by 63% (Figure 4D). Together, these data indicate that both contact-dependent and -independent mechanisms are involved in Treg expansion by histone-treated endothelial cells.

## 3. Discussion

In this study, we showed that endothelial cells activated by extracellular histones induced the expansion of a specific FoxP3^hi^ Treg subpopulation in co-culture experiments. The expression of ICAM-1 and the secretion of IL-6 by endothelial cells were involved in this expansion whereas neither PDL-1 nor IL-2 were implicated.

Extracellular histones were able to activate microvascular endothelial cells and primary renal cells revealed by the increased expression of ICAM-1 and the secretion of pro-inflammatory cytokines. This is in agreement with previous studies that showed endothelial cell activation after histone administration, in vitro and in vivo, through both TLR-dependent and -independent mechanisms [2,12,13,21]. However, the impact of extracellular histones on endothelial cell immunogenicity has not been previously studied.

Beyond their role as passive targets of inflammatory stimuli, endothelial cells can act as antigen-presenting cells. Microvascular endothelial cells express little HLA class II antigens and ICAM-1 in the steady-state; however, this expression is highly increased under inflammatory conditions [22,23,24]. They have been reported to induce T lymphocyte activation and differentiation in vitro and in vivo [25,26,27]. Indeed, previous studies in this model have shown that, under inflammatory conditions, human microvascular endothelial cells can induce proliferation of CD4^+^-T lymphocytes and selectively expand pro-inflammatory Th1 and Th17 subpopulations, whilst simultaneously expanding anti-inflammatory naïve and memory Treg subsets [28,29].

The PBMCs used for the co-culture experiments were obtained from healthy blood donors attending the local blood transfusion service. We did not find any impact of age and sex of the donors on Treg expansion in our model. We have identified certain factors controlling Treg differentiation by endothelial cells and reported the requirement for ICAM-1 and HLA-DR expression [28], the inhibition of Treg differentiation under sustained inflammatory conditions [29] or after activation of endothelial cells by pro-inflammatory mediators [30].

The differentiation of Tregs is dependent on the activation of CD4^+^-T cells by recognition of HLA II molecules expressed on the endothelial cells. This increases the variability of the responses between different donors, but this variability also strengthens the data. If the expansion of Tregs is seen to a significant level despite differential degrees of HLA-dependent activation of the primary CD4^+^-T cells by histone activated endothelial cells, this argues for a potentially important response overall in pathologies where histone release is observed. In our model, the Treg phenotype and functional assays confirmed the suppressive activity of Tregs differentiated in co-cultures of endothelial cells pre-treated with histones. Indeed, CTLA-4 is a membrane immunoglobulin functioning as an immune checkpoint and is strongly associated with Treg limitation of immune responses [31]. Moreover, the high level of expression of HLA-DR on Treg cells was reported to identify an early contact-dependent suppressive population of mature Tregs [32].

We found that ICAM-1 was involved in Treg expansion induced by histone-activated endothelial cells. This is in agreement with a previous study [28]. Indeed, Taflin et al. showed that Treg expansion by pro-inflammatory cytokine-activated endothelial-cell-required HLA-DR^+^ CD4^+^-T cell interaction with ICAM-1-expressing endothelial cells to differentiate HLA-DR^+^ functional Tregs. Bachmann et al. have shown that ICAM-1 interacts with Leukocyte Function Antigen 1 (LFA-1) on CD4^+^-T cells and enhances TCR engagement with MHC II-peptide ligand [33]. Other studies showed the importance of contact-dependent signaling in inducing memory Treg cells activation [34,35]. Decreased Treg proliferation in the absence of ICAM-1 may possibly be a result of inadequate TCR activation.

The role of IL-6 in the expansion of Tregs is more equivocal. Indeed, IL-6 was initially implicated in regulating the Th17/Treg balance, by promoting Th17, while suppressing Treg generation [36]. However, other studies have described Treg-enhancing effects of IL-6 in mice and humans [37,38,39,40,41]. Interestingly, Hagenstein et al. have shown that IL-6 induced the generation of a unique RORγt-expressing Treg subtype with enhanced suppressive capacity [41]. We have explored RORyt expression in Tregs in our model. However, while we were able to demonstrate a key role of IL-6 in the generation of suppressive Tregs, we did not find any expression of RORyt in this population. Although IL-6 has been involved in decreasing relative proportions of Tregs compared with pro-inflammatory CD4^+^-T subsets, we have recently reported that blockade of IL-6 interaction with IL-6R did not allow restoration of human Treg populations under highly inflammatory conditions [29]. The data in the current report supports the idea of an IL-6-dependent Treg population identified in this model.

Our study shows that extracellular histones induce PDL-1 expression on endothelial cells.

Expression of PDL-1 is a severity biomarker in cancer patients [42], and has been implicated in a pro-survival signal in cancer cells [43]. Upregulation of PDL-1 on endothelial cells could participate in the immunosuppressive events that reduce the therapy efficacy, particularly immunotherapies, by disrupting immune cells activation [44,45]. However, although the engagement between T-cells PD-1 and PDL-1 has been implicated in T-regs differentiation [46,47,48], PDL-1 blockade did not significantly reduce Treg cells expansion in the current model.

Although Tregs are key to the maintenance of immunologic homeostasis and tolerance, increasing interest has emerged regarding the role of Tregs in immune dysfunction. Tregs can be deleterious in cancer through suppression of anti-tumor immunity [6], and high numbers of Tregs have been correlated with a poor outcome in various malignancies [49,50,51]. In tumor lysis syndrome patients who have drastically increased levels of circulating histones [16], the increase in peripheral suppressive Treg may contribute to reduced anti-tumoral therapy efficacy.

Paradoxically, the secretion of alarmin proteins (such as extracellular histones or HMGB1) [52] by dying tumor cells has been previously shown to activate tumor antigen-specific T-cell immunity, via their action on TLR4 expressed by dendritic cells [53]. Extracellular histones may then induce opposite effects, depending on whether they act directly on dendritic cells or via their action on endothelial cell immunogenicity. The increase of peripheral suppressive Tregs via endothelial cell activation may indeed counterbalance effects on anti-tumoral immunity and reduce anti-tumoral therapy efficacy in TLS patients.

As extracellular histones have been shown to activate endothelial cells via their action on TLR4, we performed experiments using TAK242, a highly specific and well-documented TLR4 inhibitor [54] in HMECs. TLR4 inhibition did significantly inhibit the increase in peripheral suppressive Treg via endothelial cell activation induced by extracellular histones. TLR4-mediated inflammation by histones has been previously involved in experimental model of sepsis [55], acute renal injury [56] or glomerulonephritis [57]. However, some authors have found that histones were able to bind the surface of endothelial cells via their affinity for phosphodiester bonds of phospholipids, independently of their action on TLR4 [2,58,59,60]. Further studies are needed to decipher the complete mechanisms of endothelial cell activation by histones in our model.

Our study has several limitations. First, two endothelial cell types were examined. In view of the heterogeneity of endothelial cells, depending on their location [61], we cannot exclude that the immunogenicity of endothelial cells would have been different using cells from different origins. Second, as mentioned above, the exact mechanisms underlying cell activation by histones have not been fully deciphered in this study. Finally, further studies are needed to evaluate the impact on immunosuppression of an increase in peripheral suppressive Treg via endothelial cell activation by histones in vivo.

In conclusion, beyond their cytotoxic effects, we reveal a new role for extracellular histones interactions with human endothelial cells in inducing an increase of immunosuppressive Treg. Further studies are needed to investigate the role of histones on endothelial-cell-mediated adaptive immunity.

## 4. Material and Methods

### 4.1. Cell Lines and Culture Reagents

The human dermal microvascular endothelial HMEC-1 cell line, provided by A. Kesikli (University of Regensburg, Germany) was cultured in complete MCDB 131 medium, composed of MCDB 131 medium (Thermofisher Scientific, Illkirch-Graffenstaden, France) supplemented with 12.5% fetal bovine serum (FBS), hydrocortisone 1 μg/mL (Sigma-Aldrich, St. Louis, MO, USA), epidermal growth factor (EGF) 10 ng/mL (BD Biosciences, Franklin Lakes, NJ, USA), 6 mM Glutamine and used between passages 12 and 17.

Co-culture experiments were carried out with endothelial cells and peripheral blood mononuclear cells (PBMCs), as previously described [30]. Briefly, PBMCs were isolated from healthy donor blood samples (obtained in accordance with institutional regulations from the Etablissement Français du Sang) by Ficoll density gradient separation (Eurobio; les Ulysses, France). PBMCs were maintained in complete RPMI 1640 medium (Thermofisher Scientific, Illkirch-Graffenstaden, France), composed of RPMI 1640 with 10% human AB serum, HEPES 10 mM, sodium pyruvate 1 mM, and glutamine 2 mM.

For histone treatments, we had previously carried out dose-response experiments with concentrations ranging from 5 µg/mL to 40 µg/mL, according to the literature published on histones and endothelial cells. This had been published previously in Arnaud et al. [16]. We had shown that 40 µg/mL was toxic for endothelial cells and induced significant necrosis and apoptosis of HMECs. We therefore chose the concentration of 20 µg/mL that allows endothelial activation without inducing significant cell toxicity [16].

For TLR2 inhibition, HMECs were pre-incubated with anti-TLR2 antibody (50 μg/mL, Thermo Fisher Scientific, Illkirch-Graffenstaden, France) for 1 h prior to histone stimulation. Mouse IgG2a, k antibody (50 μg/mL, Thermo Fisher Scientific, Illkirch-Graffenstaden, France) was used as an isotype control.

For TLR4 inhibition, HMECs were pre-incubated with the TAK-242 TLR4 inhibitor (Tocris, Biotechne, Bristol, UK), added for 1 h at 1 µM prior to histone stimulation.

### 4.2. Co-Culture Assays

Human endothelial cells were washed and seeded to produce a confluent monolayer before addition of extracellular histones (20 µg/mL) (Sigma Aldrich, St. Louis, MO, USA) and left overnight. IFNγ was added at a concentration of 3 ng/mL in order to induce HLA-DR expression.

Where indicated, the TAK-242 TLR4 inhibitor (Tocris, Biotechne, Bristol, UK) was added for 1 h at 1 µM prior to histones. Where indicated, endothelial cells were incubated with the indicated concentrations of blocking antibodies for 1 h at 37 °C (monoclonal Ab includes anti-IL6, anti-ICAM1, anti-PDL1 (BioLegend, San Diego, CA, USA)) before removing the antibody solution and washing with PBS. Where indicated, co-cultures were performed in Transwell^®^ inserts (0.4 µm, Corning, Kennebunk, ME, USA) in the same conditions.

Human endothelial cells were cultured with PBMCs (1:1) for 3 days. Treg subsets were identified by flow cytometry at day 3 (see below), and samples of co-culture supernatants were taken for the analysis of soluble factors.

### 4.3. Flow Cytometry and Antibodies

Endothelial cells were phenotyped using Human Leucocyte Antigen (HLA)-DR APC, ICAM-1 AF488, CD106 PE-Cy7, CD137 Pacific Blue, CD62-P PerCP-Cy5.5, HLA-ABC APC-Cy7, CD31 AF488, CD274 BV510.

For CD4^+^ T subsets, the following antibodies were used: CD4 PE, CD3 PerCP, and intracellular staining of IFNγ FITC and IL-17 APC was used for the discrimination of the Th1 and Th17 subsets, respectively. Tregs were discriminated with CD45RA PE-Cy7, CD25 PE, CD127 PerCP-Cy5.5 and intracellular staining of FoxP3 BV421 using the Human Foxp3 Staining Set (236A/E7; eBioscience, San Diego, CA, USA).

Treg subsets were identified as memory Treg CD4^+^ CD45RA^−^ FoxP3^hi^, naïve Treg CD4^+^ CD45RA^+^ FoxP3^high^. Two other CD4^+^ T lymphocyte subsets were studied: Th17 CD3^+^ CD8^−^ IL-17^+^ or Th1 CD3^+^ CD8^−^ IFNγ^+^. Characterization of Treg phenotype was also assessed with HLA-DR PE, CTLA-4 PE-Cy7 et RORyt BV510.

Gating was performed using Fluorescence Minus One (FMO) control. Flow cytometry was carried out on a FACS Canto II (BD).

### 4.4. Treg Suppression Assay

PBMCs were prepared from fresh donor blood or thawed, then co-cultured as described above. We used methods previously described in the literature [18,19] for the following protocol. Detailed protocol is provided in Supplementary Figure S6.

Tregs from our model were stained with CD4 FITC, CD25 PE, and CD127 PerCP-Cy5.5 and sorted on a BD FACS Aria II system.

In parallel, autologous PBMCs were cultured in T25 flasks and CD4^+^ T cells were sorted the same day as Tregs on BD FACS Aria II, with a CD4 FITC prior staining with CellTrace Violet proliferation kit (ThermoFisher, Illkirch-Graffenstaden, France). These cells are labeled as responders T cell (Tresp).

Sorted Tregs and stained Tresp were then put in coculture together at different ratio for 3 days. We added Dynabeads T activator CD3/CD28 (ThermoFicher; Illkirch-Graffenstaden, France), 1/10 diluted. Proliferation of Tresp cells was analyzed by flow cytometry on BD FACS CantoII.

### 4.5. Cytokine Quantification

The following cytokines were quantified by enzyme-linked immunosorbent assay according to the manufacturer’s protocol: human IL-6, IL-10, IL-2, MCP-1 (Biolegend, San Diego, CA, USA).

### 4.6. Statistical Analysis

Statistical analysis was performed using the GraphPad Prism software 7 (GraphPad Software, San Diego, CA, USA). The results are reported as mean ± standard error of the mean (SEM). Normality was assessed using Shapiro–Wilk test. Differences between groups were analyzed for statistical significance by t-test or one-way analysis of variance (ANOVA) for repeated measures and subsequent Bonferroni post hoc test. Non-normally distributed single measurements were compared using the Mann–Whitney U test or Kruskal–Wallis test with Dunn post hoc test. A *p* < 0.05 was considered statistically significant.

## Data Availability

The data presented in this study are available on request at marine.arnaud@inserm.fr.

## Supplementary Materials

The following supporting information can be downloaded at: https://www.mdpi.com/article/10.3390/ijms23094527/s1.
