# Supplementary material for: Endothelial Cells Activated by Extracellular Histones Promote Foxp3+ Suppressive Treg Cells In Vitro
Source: Int J Mol Sci. 2022 Apr 20;23(9):4527. doi: 10.3390/ijms23094527 (PMC9103825; doi:10.3390/ijms23094527)
Supplement: Supplementary file 1 [file ijms-23-04527-s001.zip › ijms-1665285-supplementary.pdf]

Type of paper: *Brief reports*

Title: Endothelial cells activated by extracellular histones promote FoxP3⁺ suppressive Treg cells *in vitro*

Supplemental data

Figure S1: Effect of histones H1, H2A, H3 and H4 on HMECs

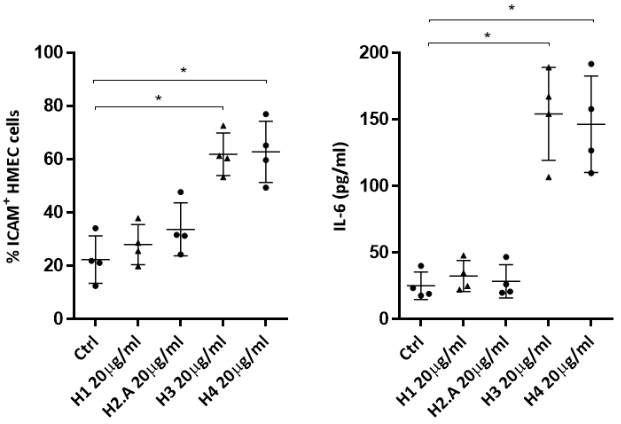

ICAM-1 was assessed in HMEC cells activated with 20 µg/mL of purified individual histones (H1, H2.1, H3, H4). Percentage of positive cells were evaluated by cytometry. Levels of IL-6 in the supernatant were measured by ELISA. Kruskal-Wallis test,**p*=0.006, n=4

Figure S2: Anti-TLR2 effect on endothelial cells expression of ICAM-1, PDL-1 and secretion of IL-6

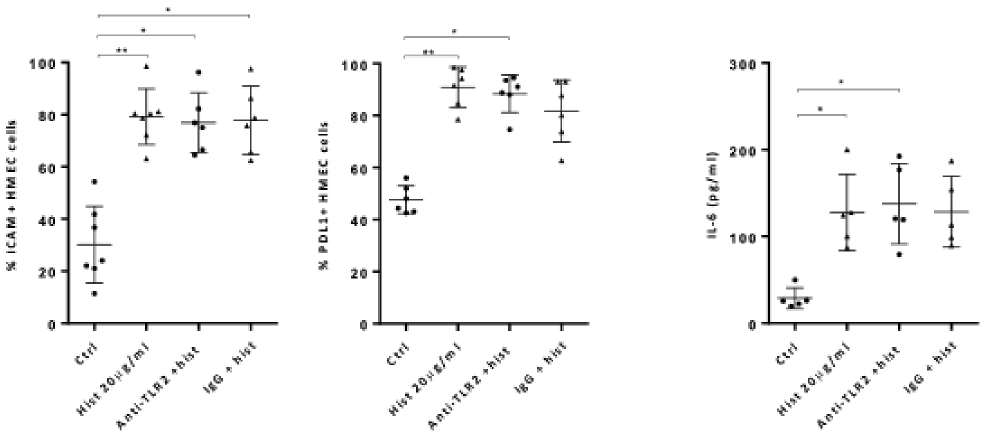

CD54 (ICAM-1) and CD106 (Vascular cell adhesion protein 1 (VCAM-1)) expression were measured on Human Microvascular Endothelial Cells (HMEC) after treatment with 20 µg/mL

histone, with or without IgG anti-TLR2 50 µg/mL or a control IgG antibody 50 µg/mL. Median Fluorescence Intensity (MFI) and percentage of positive cells were measured by cytometry. Kruskal-Wallis test,****p*=0,002, n = 6

InterLeukin-6 was measured by ELISA in the supernatant. Kruskal-Wallis test,***p*=0.013, n=5

Figure S3: Effect of sex and age of PBMCs donors on Treg expansion

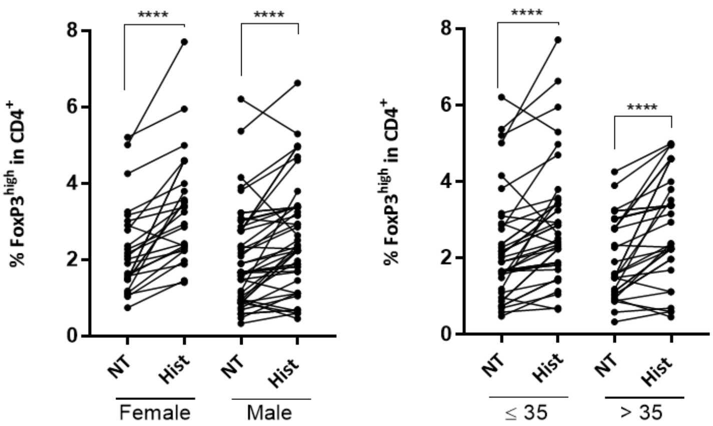

Comparison of Treg expansion after 3-days of coculture, with or without histone pre-stimulation of HMECs. Left panel are results presented as percentage of $CD4^+$ $CD25^+$ $CD127^{low}$ $FoxP3^{high}$ among $CD4^+$ cells according to the sex of the PBMCs donors. Wilcoxon test, *****p*<0,0001

Right panel are results presented as percentage of $CD4^+$ $CD25^+$ $CD127^{low}$ $FoxP3^{high}$ among $CD4^+$ cells according to the age of the PBMCs donors. Age ≤ 35 years old, n=40; age > 35 years-old, n=33. 35 years-old corresponds to the median age of the PBMCs donors. Wilcoxon test, *****p*<0,0001.

Figure S4: Gating strategy for Treg cells identification and phenotype

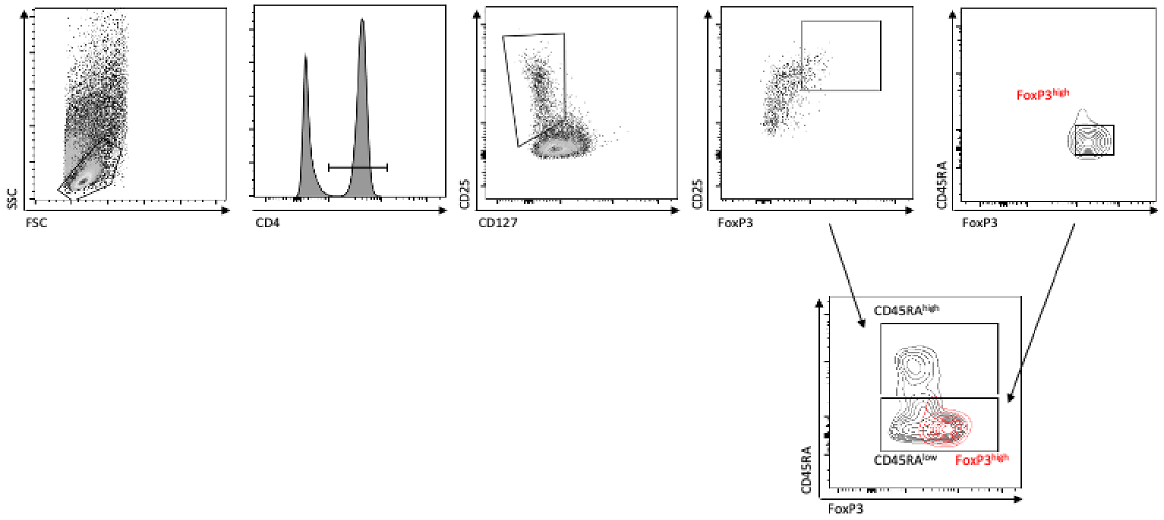

Gating strategy to identify Treg cells after 3 days of coculture with HMEC stimulated with histone for 18h. Treg cells are CD4$^+$ CD25$^+$ CD127^{low} FoxP3^{high} T lymphocytes, memory Treg are CD45$^-$ and naïve Treg cells are CD45RA$^+$. Gating was performed using Fluorescence Minus One (FMO) control.

Figure S5: Th17, Th1 and Treg RORγT$^+$ population at the end of 3-days cocultures

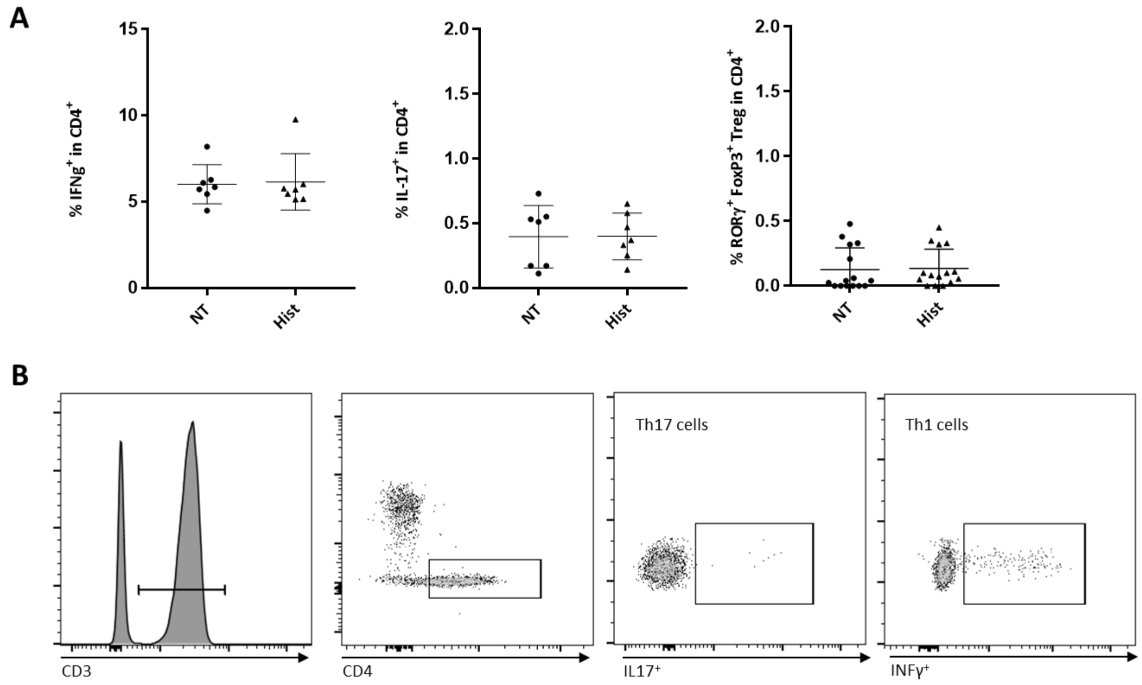

A/ Comparison of Th17, Th1 and RORγt-$Treg^{+}$ expansion after 3 days of coculture, with or without histone pre-stimulation of HMECs. Results are presented as percentages of respectively $CD3^{+}$ $CD4^{+}$ IL-17^{+} and $CD3^{+}$ $CD4^{+}$ $IFNγ^{+}$ among $CD4^{+}$ cells. n=7 for Th1 and Th17 cells, n=30 for $RORγt^{+}$ Tregs cells. Paired t test (ns).

B/ Gating strategy for Th1 and Th17 cells. FoxP3 $RORγt^{+}$ are defined as $CD4^{+}$ $CD25^{+}$ $CD127^{low}$ $FoxP3^{high}$ $RORγt^{+}$.

Figure S6: Suppression assay method

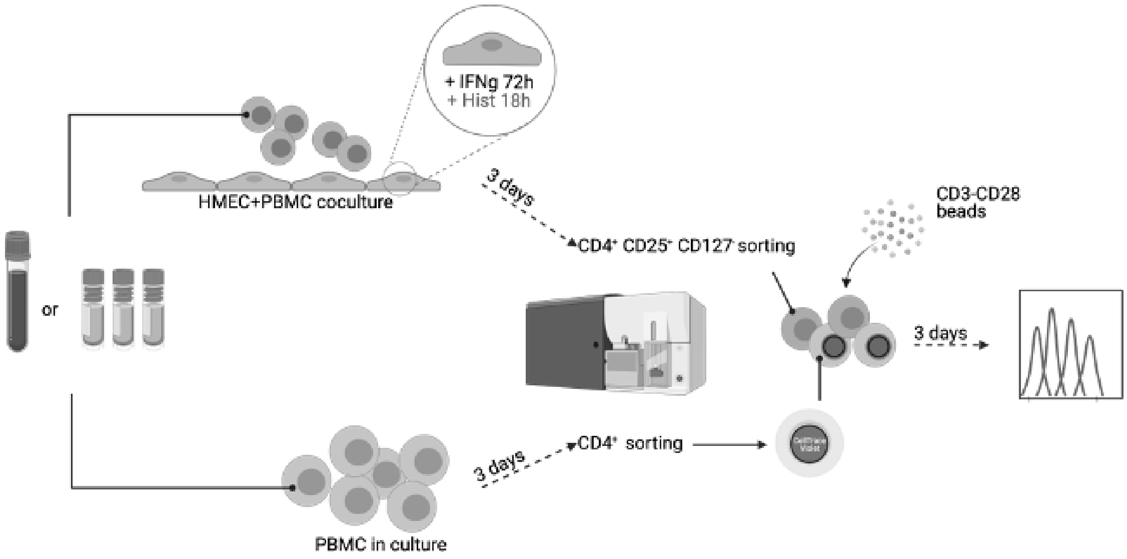

PBMCs were prepared from fresh donor blood or thawed, and cocultured as described above. We used methods previously described in literature [17], [52] for the following protocol. Treg cells are generated in our in vitro model, for 3 days in cocultures with histone-stimulated endothelial cells. Tregs were stained with CD4 FITC, CD25 PE, and CD127 PerCP-Cy5.5 and sorted on a BD FACS Aria II system. In parallel, autologous PBMCs were cultured in T25 flasks and $CD4^{+}$ T cells were sorted the same day as Tregs on BD FACS Aria II, with a CD4 FITC prior staining with CellTrace Violet proliferation kit (ThermoFisher). These cells are considered as responders T cell (Tresp). Sorted Tregs and stained Tresp were then cocultured together at different ratio for 3 days. We added Dynabeads T activator CD3/CD28 (ThermoFicher), 1/10 diluted. Proliferation of Tresp cells was analyzed by flow cytometry on BD FACS CantoII.

Table S1: HMEC1 phenotype

Percentage of HMEC positive cells		NT	Hist	IFNg -	IFNg Hist
Adhesion molecules	CD31	55,3	55,5	61,45	63,15
	CD54	3,07	21,7	49,9	59,5
HLA molecules	HLA-DR	0,00861	0,018	88,7	89,2
	HLA-I	95,8	96,6	81,5	75,3
Co-stimulatory molecules	CD274	0,69	12,5	54,3	57,7

HMEC1 cells phenotype without any treatment (NT), with or without Interferon (IFN)γ at 30ng/ml for 3 days and with or without extracellular histones (hist) at 20µg/ml during 18h. Results are presented as percentage of positive cells for each marker. VCAM, 4-1 BBL, CD62P were not detectable by cytometry on HMEC1 cells.
